# Loss of Maternal CTCF Is Associated with Peri-Implantation Lethality of *Ctcf* Null Embryos

**DOI:** 10.1371/journal.pone.0034915

**Published:** 2012-04-20

**Authors:** James M. Moore, Natalia A. Rabaia, Leslie E. Smith, Sara Fagerlie, Kay Gurley, Dmitry Loukinov, Christine M. Disteche, Steven J. Collins, Christopher J. Kemp, Victor V. Lobanenkov, Galina N. Filippova

**Affiliations:** 1 Human Biology Division, Fred Hutchinson Cancer Research Center, Seattle, Washington, United States of America; 2 Molecular Pathology Section, Laboratory of Immunopathology, National Institute of Allergy and Infectious Diseases, National Institutes of Health, Rockville, Maryland, United States of America; 3 Department of Pathology, University of Washington, Seattle, Washington, United States of America; National University of Singapore, Singapore

## Abstract

CTCF is a highly conserved, multifunctional zinc finger protein involved in critical aspects of gene regulation including transcription regulation, chromatin insulation, genomic imprinting, X-chromosome inactivation, and higher order chromatin organization. Such multifunctional properties of CTCF suggest an essential role in development. Indeed, a previous report on maternal depletion of CTCF suggested that CTCF is essential for pre-implantation development. To distinguish between the effects of maternal and zygotic expression of CTCF, we studied pre-implantation development in mice harboring a complete loss of function *Ctcf* knockout allele. Although we demonstrated that homozygous deletion of *Ctcf* is early embryonically lethal, in contrast to previous observations, we showed that the *Ctcf* nullizygous embryos developed up to the blastocyst stage (E3.5) followed by peri-implantation lethality (E4.5–E5.5). Moreover, one-cell stage *Ctcf* nullizygous embryos cultured *ex vivo* developed to the 16–32 cell stage with no obvious abnormalities. Using a single embryo assay that allowed both genotype and mRNA expression analyses of the same embryo, we demonstrated that pre-implantation development of the *Ctcf* nullizygous embryos was associated with the retention of the maternal wild type *Ctcf* mRNA. Loss of this stable maternal transcript was temporally associated with loss of CTCF protein expression, apoptosis of the developing embryo, and failure to further develop an inner cell mass and trophoectoderm *ex vivo*. This indicates that CTCF expression is critical to early embryogenesis and loss of its expression rapidly leads to apoptosis at a very early developmental stage. This is the first study documenting the presence of the stable maternal *Ctcf* transcript in the blastocyst stage embryos. Furthermore, in the presence of maternal CTCF, zygotic CTCF expression does not seem to be required for pre-implantation development.

## Introduction

CTCF (CCCTC-binding factor) is a highly conserved, ubiquitously expressed 11 Zn finger DNA binding protein that was originally identified as a factor interacting with specific sequences in the *c-myc* gene promoters [1], [2], [3]. CTCF utilizes different combinations of its Zn- fingers to bind a relatively large number of highly divergent target sequences throughout genome [3], [4], [5], [6], [7]. CTCF was originally characterized as a transcription factor involved in repression [2], [3], [8], [9] or activation of transcription [10], [11], but its activity has now been extended to include a variety of other roles in gene regulation, including chromatin insulation [4], [12], [13]. Indeed, genomic regions displaying chromatin insulator activity harbor CTCF binding sites, and CTCF binding is required for their insulator activity [6], [14], [15], [16]. Moreover, CTCF binding has been shown to mark boundaries between distinct chromatin domains in the genome [6], [17], [18]. Methylation-sensitive CTCF binding plays a critical role in regulating genomic imprinting at several genomic loci, including the *Igf2/H19* locus [19], [20], [21], [22]. In addition CTCF plays an important role in X-chromosome inactivation [23], [24], and genes which escape X-inactivation are separated from stably inactivated X-chromosome genes by CTCF binding sites [25]. CTCF has also been shown to be involved in organizing higher order chromatin structure including the interchromosomal association of transcriptionally active genes [12], [13], [26].

Given the multifunctional nature of CTCF in regulating gene expression and a recent report on the essential function of CTCF in pre-implantation development using the oocyte-specific RNAi approach [27], we wished to determine what effect a complete loss of zygotic CTCF expression would have on embryogenesis. Accordingly, we engineered mice harboring a complete loss of function *Ctcf* allele. While mice heterozygous for this allele appeared phenotypically normal, nullizygous mice displayed early embryonic lethality at the peri-implantation stage. Interestingly, *Ctcf* nullizygous embryos appeared morphologically normal up to the blastocyst stage of development, which was associated with expression of the maternal wild type *Ctcf* transcript and protein. Subsequent loss of this stable maternal transcript was temporally associated with loss of CTCF protein expression, apoptosis and embryonic lethality.

## Results

### Generation of *Ctcf* (+/−) mice

**Figure 1:**
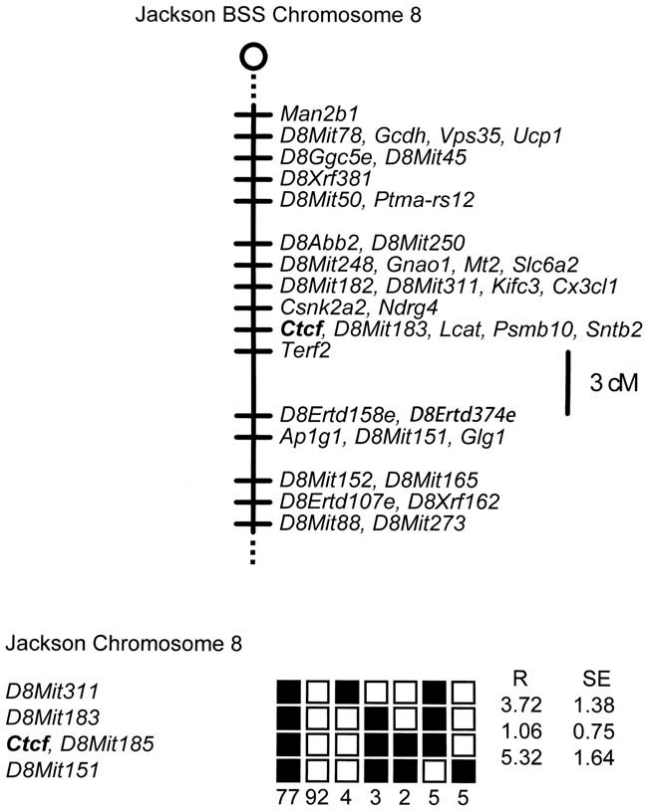
*Ctcf* maps to mouse chromosome 8. The Jackson Laboratory interspecific backcross panels (BSS and BSB) [28] were utilized to map the *Ctcf* locus to mouse chromosome 8. The loci are listed in order with the most proximal at the top. The black boxes represent the C57BL6/JEi allele and the white boxes the SPRET/Ei allele. The number of animals with each haplotype is given at the bottom of each column of boxes. The percent recombination (R) between adjacent loci is given to the right of the lower figure together with the standard error (SE) for each R.

**Figure 2:**
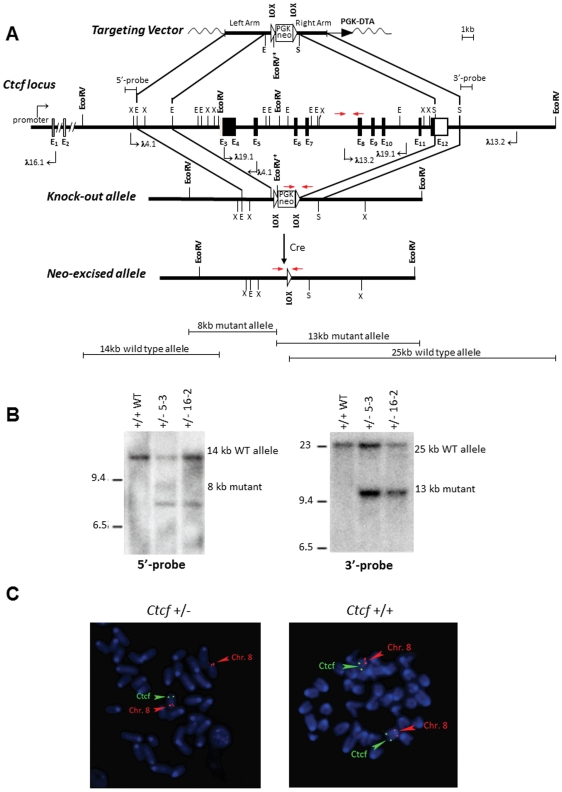
Generation of *Ctcf* knockout mice. (**A**) A schematic diagram of the mouse *Ctcf* locus and the targeting vector derived from the wild type *Ctcf* allele as described in Materials and Methods are shown. E_1_–E_12_ denote *Ctcf* exons 1 through 12. Restriction enzyme sites shown on map are as follows: E denotes *Eco*RI, X denotes *Xba*I, and S denotes *Spe*I. The locations of both the 5-prime and 3-prime *Ctcf* genomic probes for the Southern blot analysis are indicated. Red arrows show the locations of genotyping primers. A schematic diagram of genomic digest with *Eco*RV of the *Ctcf* knock-out and wild type alleles is shown below. (**B**) A Southern blot analysis of *Eco*RV digested genomic DNA utilizing the 5-prime and 3-prime *Ctcf* genomic probes distinguishes the wild type (14 kb or 25 kb) from the mutated (8 kb or 13 kb) *Ctcf* alleles. (**C**) FISH analysis of chromosome spreads from lymphocyte cultures derived from *Ctcf* heterozygous (+/−) and wild type (+/+) mice. The probe for *Ctcf*, a lambda phage clone 19.1 containing a 17 kb *Ctcf* genomic DNA insert that includes all coding *Ctcf* exons that were deleted in the *Ctcf* knockout allele (Figure 2A), was visualized with fluorescein (green fluorescence). The probe for chromosome 8 identification, a BAC clone (MB11301, Research Genetics), was visualized with Cy3 (red fluorescence). Examples of single metaphase cells from the *Ctcf* (+/−) and *Ctcf* (+/+) mice after hybridization to the *Ctcf* and chromosome 8 probes are shown. Both homologues of chromosome 8 are labeled with a red and a green signal in the wild type mouse, whereas absence of green signals on one homologue of chromosome 8 confirmed the presence of a deletion of *Ctcf* in the *Ctcf* (+/−) mouse.

We previously observed that the coding region of *CTCF* exhibits marked (>93%) evolutionary conservation in chickens, mice and humans [3], and we therefore utilized a human *CTCF* cDNA probe to clone the mouse *Ctcf* genomic locus. Utilizing the Jackson Laboratory Interspecific Backcross DNA Panels [28] together with a polymorphic CAA repeat marker that we identified in the second intron of the mouse *Ctcf* locus, we mapped *Ctcf* to a single locus in distal mouse chromosome 8 (Figure 1). Subsequently we observed that the *Ctcf* locus in mouse (Figure 2A) and human [5], [29] has a virtually identical exon/intron structure consisting of two non-coding and ten coding exons.

Our efforts to engineer *Ctcf* knockout mice via homologous recombination in mouse embryonic stem (ES) cells are detailed in Materials and Methods. We first isolated the mouse *Ctcf* genomic locus from a 129sv mouse genomic library, engineered a targeting vector from the *Ctcf* genomic clones and used this vector to generate a null allele (Figure 2A). We chose to generate an allele that deleted all coding exons of *Ctcf* since only partial deletion may result in production of an aberrant CTCF protein arising from internal cryptic translation initiation sites and alternative splicing.

The linearized targeting vector was electroporated into ES cells, and positive recombination was scored by Southern blots with the designated 5-prime and 3-prime probes located outside of the targeting cassette (Figure 2A, B). Seven positive ES clones were selected to produce chimeric mice and two clones of *Ctcf* (+/−) heterozygous knockout mice were established, as confirmed by the presence of both the wild type and deleted *Ctcf* alleles documented by Southern blot hybridization of *Eco*RV-digested tail genomic DNA (Figure 2B). To excise the neo cassette, one clone (5-3) of *Ctcf* (+/−) heterozygous knockout mice was crossed to the MORE (Mox2Cre) mice, which express the Cre recombinase from the *Mox2* locus [30]. The resulting neo-excised *Ctcf* allele was documented by PCR using primers flanking the remaining LOX site shown in Figure 2A.

Deletion of the *Ctcf* locus in the *Ctcf* knockout allele was also confirmed utilizing a FISH assay on metaphase chromosomes from lymphocyte cultures derived from *Ctcf* (+/−) and *Ctcf* (+/+) mice (Figure 2C). As a *Ctcf* probe we utilized a 17 kb lambda *Ctcf* genomic clone (19.1) containing all of the coding exons that were deleted in the *Ctcf* knockout allele (Figure 2A). As a positive control we used a centromeric probe from mouse chromosome 8 where *Ctcf* maps (170 kb BAC clone MGB 11301). The *Ctcf* probe was visualized with fluorescein (green) and centromeric probe for chromosome 8 was visualized with Cy3 (red). Only a single chromosome from the *Ctcf* (+/−) metaphase spreads displayed a *Ctcf* signal, while two signals were clearly seen in similar spreads from the *Ctcf* (+/+) mice (Figure 2C).

### *Ctcf* knockout mice exhibit embryonic lethality

**Figure 3:**
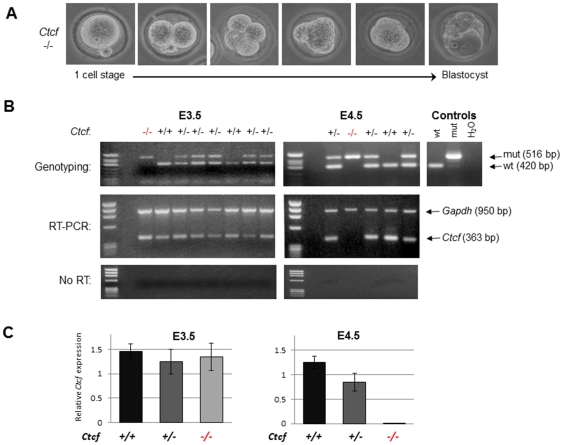
*Ctcf* (−/−) embryos retain maternal *Ctcf* mRNA and develop to the blastocyst stage. (**A**) E0.5 one-cell stage embryos were isolated from *Ctcf* (+/−) heterozygous intercrosses and cultured *ex vivo* as detailed in Materials and Methods. (**B**) A single embryo assay shows genotyping and RT-PCR analysis of DNA and RNA simultaneously isolated from the same freshly isolated E3.5 and E4.5 pre-implantation stage embryos. The top panel shows a representative multiplex PCR-based genotyping analysis of genomic DNA from these embryos, which distinguishes wild type and mutated *Ctcf* alleles. The next panel shows multiplex RT-PCR analysis of mRNA from the same pre-implantation embryos for the presence of *Ctcf* and *Gapdh* transcripts. The bottom panel shows ‘no RT’ controls for the same samples. The displayed results are representative of over 60 pre-implantation embryos that were analyzed from intercrosses of *Ctcf* (+/−) heterozygous mice. *Hae*III digested ϕX DNA was utilized as DNA molecular weight markers in these gels. (**C**) Real time RT-PCR analysis of *Ctcf* expression was performed on genotyped E3.5 and E4.5 embryos from *Ctcf* (+/−) heterozygous intercrosses. *Ctcf* RNA levels were normalized to *Gapdh* expression. The results represent the mean of 3–6 independent embryos of the indicated genotype. Each embryo was assayed in triplicate, data represents mean +/− standard error.

**Figure 4:**
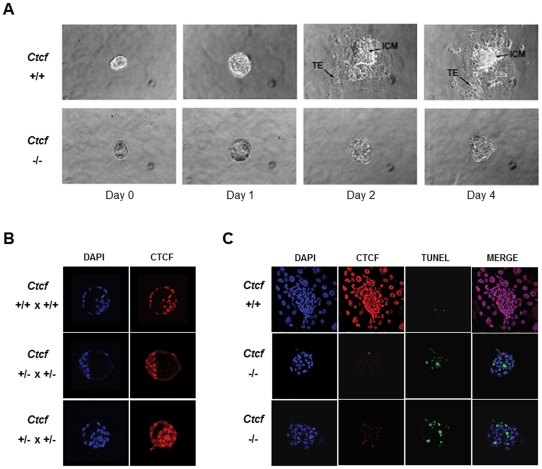
*Ctcf* nullizygous embryos fail to develop beyond the E3.5 stage and undergo apoptosis. (**A**) *Ex vivo* outgrowth of E3.5 embryos. Blastocysts (E3.5) were isolated from *Ctcf* heterozygous (+/−) intercrosses and cultured *ex vivo* for the indicated number of days. At the end of the culture period the embryos were harvested and PCR genotyping and RT-PCR performed. The cultured *Ctcf* (+/+) and (+/−) embryos outgrew to form an inner cell mass (ICM) and trophoectoderm (TE) while little proliferation was noted in the cultured *Ctcf* (−/−) embryos (40×). (**B**) E3.5 blastocyst stage embryos isolated from the indicated crosses were subjected to DAPI staining and anti-CTCF immunochemistry (40×). All (16/16) embryos analyzed from the *Ctcf* (+/−) heterozygous intercrosses were positive for CTCF protein expression, displaying staining patterns similar to the two representative embryos shown here. The expected Mendelian ratio of genotypes in such crosses indicates that the chance that none of these 16 embryos were *Ctcf* (−/−) is less than 0.05 (i.e. (3/4)^16^≅0.01). (**C**) Day two outgrowths of E3.5 embryos of the indicated genotype. Blastocysts (E3.5) were cultured *ex vivo* for two days and then subjected to DAPI staining, anti-CTCF immunohistochemistry and TUNEL assay (40×). *Ctcf* (+/+) and (+/−) embryos outgrew and expressed CTCF, while cultured *Ctcf* (−/−) embryos failed to outgrow and exhibited loss of CTCF protein expression and increased apoptosis. The observations displayed here involve one *Ctcf* (+/+) and two *Ctcf* (−/−) embryos cultured *ex vivo* and are representative of over 60 embryos that were analyzed from *Ctcf* (+/−) heterozygous intercrosses.

To study the effect of CTCF knockout on embryonic development, we generated the *Ctcf* (+/−) heterozygous mice by crossing C57/BL6 *Ctcf* (+/+) mice to 129sv *Ctcf* (+/−) mice. The C57BL6/129sv F1 C*tcf* (+/−) mice were viable and displayed no apparent phenotypic abnormalities. However, when intercrossing heterozygotes we observed a selective absence of *Ctcf* (−/−) pups in the newborn offspring (Table 1, top row) indicating that the absence of functional CTCF is lethal to the developing embryo. To determine at what stage of embryonic development this lethality occurs we genotyped embryos obtained at different stages of development following the breeding of *Ctcf* (+/−) heterozygotes. For this genotyping we utilized a multiplex PCR- based assay that distinguishes the wild type from mutant *Ctcf* alleles (Figure 3B). We observed a Mendelian ratio of genotypes at embryonic day 3.5 (E3.5) (Table 1). *Ctcf* (−/−) E3.5 blastocysts had a distinct blastoceal and intact zona pellucida, displaying no phenotypic abnormalities compared with their wild type or heterozygous littermates (Figure 4A). However, no *Ctcf* (−/−) embryos were observed at embryonic day 5.5 (E5.5) and beyond (Table 1). Notably, the number of empty deciduae in E5.5–6.5 heterozygous intercrosses was markedly increased in comparison to control crosses (Table 2). Taken together our data indicate that the *Ctcf* (−/−) embryos fail to implant and become non-viable between E4.5 and E5.5.

**Table 1:** *Ctcf* nullizygous mice display early embryonic lethality.

Cross	Stage	Average number of pups or embryos per cross	*Ctcf* genotype		
Female×Male			+/+	+/−	−/−
(+/−)×(+/−)	Postnatal	7.2	39	84	0
(+/−)×(+/+)*	Postnatal	7.1	29	27	NA
(+/+)×(+/−)*	Postnatal	7.4	18	19	NA
(+/−)×(+/−)	E12.5	7.5	4	11	0
(+/−)×(+/−)	E10.5	8.1	8	16	0
(+/−)×(+/−)	E8.5	7.6	13	26	0
(+/−)×(+/−)	E6.5	8	10	24	0
(+/−)×(+/−)	E5.5	7	6	14	0
(+/−)×(+/−)	E3.5	8.5	18	34	14

* Control crosses.

NA = not applicable.

**Table 2:** Analysis of E5.5 and E6.5 *Ctcf* embryos.

Cross		Total # of deciduae	*Ctcf* genotype of embryos			# of empty deciduae
Female	Male		+/+	+/−	−/−	
Heterozygous crosses						
+/−	+/−	72	16	38	0	18
Control crosses						
+/−	+/+	15	6	9	NA	0
+/+	+/−	23	7	15	NA	1

NA = not applicable.

### *Ctcf* (−/−) embryos reach the blastocyst stage but fail to develop further, which is associated with the loss of the maternal *Ctcf* mRNA and CTCF protein

To gain further insight into the embryonic defects resulting from the absence of CTCF we isolated newly fertilized eggs from *Ctcf* heterozygous crosses, cultured these embryos *ex vivo* and then genotyped them. Cultures of explants from E0.5 one cell stage *Ctcf* (+/+), (+/−) and (−/−) embryos all underwent cell division with many embryos successfully reaching the 32 cell blastocyst stage within four days (Figure 3A and Table 3). We observed no gross phenotypic abnormalities in the cultured pre-implantation stage *Ctcf* (−/−) embryos compared with their wild type or heterozygous littermates.

**Table 3:** *Ctcf* nullizygous embryos can develop *ex vivo* to the morula/blastocyst stage.

*Ctcf* genotype	1–8 cell	Morula/Blastocyst	Total
+/+	6	7	13
+/− or −/+	10	18	28
−/−	5	4	9

To confirm the absence of CTCF expression in the *Ctcf* (−/−) E3.5 blastocyst stage embryos, we developed a single embryo assay that allowed both genotyping and mRNA expression analysis of the same embryo as detailed in Materials and Methods. For both genotyping and RT-PCR, we utilized multiplex PCR conditions to control for quality of genomic DNA and cDNA, respectively. Unexpectedly, RT-PCR analysis of mRNA extracted from E3.5 blastocyst stage embryos isolated from *Ctcf* (+/−) intercrosses revealed *Ctcf* transcripts not only in the *Ctcf* (+/+) and (+/−) embryos but also in the *Ctcf* (−/−) embryos (Figure 3B, left panel). In contrast, we could not detect any *Ctcf* transcripts in the freshly isolated E4.5 stage *Ctcf* (−/−) embryos (Figure 3B, right panel). These observation were confirmed utilizing a quantitative real-time RT-PCR assay of the *Ctcf* (+/+), (+/−), and (−/−) embryos (Figure 3C). The presence of the *Ctcf* mRNA in the *Ctcf* (−/−) blastocyst stage embryos likely represents the retention in the developing embryo of the maternal oocyte *Ctcf* transcripts that appear to remain up to E3.5 stage of development but are lost by E4.5. Although we found it technically difficult to perform both *Ctcf* mRNA quantitation and anti-CTCF immunohistochemistry on the same embryo, we observed that all (16/16) E3.5 embryos from *Ctcf* heterozygous intercrosses were positive by immunohistochemistry for CTCF protein expression (Figure 4B). The chance that none of these 16 embryos were *Ctcf* (−/−) according to the expected Mendelian ratio of genotypes is less than 0.05. This strongly suggests that the *Ctcf* mRNA present in the E3.5 *Ctcf* (−/−) embryos is indeed associated with CTCF protein expression.

To gain further insight into the mechanism of early embryonic lethality of the *Ctcf* (−/−) embryos, we isolated E3.5 blastocyst stage embryos from *Ctcf* heterozygous intercrosses and cultured them *ex vivo*. The cultured embryos were then PCR genotyped and assessed for *Ctcf* transcript expression utilizing RT-PCR. We observed that while the *ex vivo* cultures of the *Ctcf* (+/+) and (+/−) E3.5 stage embryos actively proliferated over 2–4 days to form an inner cell mass and trophoectoderm (Figure 4A, top panel), the *Ctcf* (−/−) E3.5 embryos explants failed to outgrow and the inner cell mass and trophoectoderm did not develop (Figure 4A, bottom panel). While both *Ctcf* transcripts and CTCF protein were observed in the *Ctcf* (−/−) E3.5 blastocyst stage embryos (Figure 3B,C and Figure 4B), following 2 days of *ex vivo* culture these embryos no longer expressed *Ctcf* mRNA or protein (data not shown and Figure 4C). Apoptosis as assessed by TUNEL assay was much more pronounced in the cultured *Ctcf* (−/−) blastocysts compared with the cultured *Ctcf* (+/+) blastocysts (Figure 4C).

## Discussion

The ubiquitous expression of CTCF, its marked evolutionary conservation and its central role in controlling gene expression by regulating DNA methylation, genomic imprinting, insulator activity, and interchromosomal associations [4], [5], [12], [13] suggests that it may be a crucial regulator of cell viability, proliferation, and differentiation. This is indeed confirmed in the present study in which we observe that mouse embryos nullizygous for *Ctcf* become arrested in development very early in embryogenesis. Embryos undergo initial cell division and reach the blastocyst stage (E3.5). However, these *Ctcf* nullizygous embryos appear incapable of uterine implantation and invariably undergo extensive apoptosis between E4.5 and E5.5.

Given the recently documented essential role for CTCF in pre-implantation development [27], [31], together with the previous observation that conditional loss of CTCF expression in cell culture is associated with the rapid onset of apoptosis [32], [33], it is perhaps surprising that the *Ctcf* nullizygous embryos are even capable of repeated cell division to reach the 32 cell blastocyst stage. We clearly demonstrate here that this initial cell proliferation/viability exhibited by the *Ctcf* (−/−) embryos is associated with the retention of the wild type maternally-derived *Ctcf* transcript at the preimplantation stage. Maternal transcripts in general are well documented to be present in the embryo up to the two-cell stage, when the zygotic transcription is activated, however recent evidence suggests that certain maternal transcripts are still retained up to the blastocyst stage [34], [35], [36]. In fact, many embryonically lethal null mutations are believed to be masked by prolonged maternal expression of the genes and therefore rarely result in early cleavage-stage pre-implantation lethality [37], [38], [39]. Indeed, we reproducibly detected *Ctcf* maternal transcripts in the *Ctcf* nullizygous blastocysts corresponding to E3.5 (Figure 3B,C), suggesting that the presence of this wild type transcript may be involved in maintaining the viability of the *Ctcf* (−/−) embryo during pre-implantation development. Consistent with this hypothesis, we observed that by E4.5 stage of development, the *Ctcf* maternal transcript is no longer detected in the *Ctcf* nullizygous embryos, and this is temporally associated with the onset of apoptosis.

In mammals, the transition between oocyte and embryo development occurs in the absence of active transcription and depends on the presence and coordinated translation of stored mRNAs transcribed in the growing oocyte [40], [41], [42]. The stability and translation of such maternal transcripts was found to correlate with the presence of cytoplasmic polyadenylation elements (CPE) within about 90–120 nucleotides 5-prime of the nuclear polyadenylation signal, AAUAAA [43]. Consistently, cytoplasmic polyadenylation of some maternal transcripts was shown to be necessary for oocyte maturation and initiation of pre-implantation development in the mouse [44]. Several RNA-binding proteins including the CPE-binding protein (CPEB) were shown to bind CPEs to form a translation-repressing complex that upon activation would release the mRNA allowing formation of the initiation complex [41], [45], [46]. The CPE was originally identified as an UUUUAU sequence in 3-prime UTRs of maternal transcripts. Survey of 3-prime UTRs of a series of maternal mRNAs that are relatively abundant in the mouse oocyte and zygote expanded this consensus to (A)UUUU(UU)A(UAA) [43]. In our study, we identified a stable maternal *Ctcf* mRNA that is efficiently translated and present in the embryo up to the implantation stage, suggesting that *Ctcf* transcripts might have a potential CPE element in its 3-prime UTR. Indeed, a CPE-like sequence, AUUAUUUUA, is located ∼80 nt upstream from a classic nuclear polyadenylation signal AAUAAA in the mouse *Ctcf* mRNA. Moreover, this potential CPE in *Ctcf* 3-prime UTR is 100% conserved between mouse (NCBI Accession Number: NM_181322) and human (NCBI Accession Number: NM_006565).

Maternal-effect genes are usually transcribed in oocytes and essential for early embryonic development [47], [48], [49], [50]. Chromatin structure has been shown to be important for reprogramming gene expression during zygotic genome activation (ZGA) in pre-implantation development [38], [51], [52], [53]. Perhaps it is not surprising that CTCF that is involved in chromatin insulation and organization of higher order chromatin structure was found to be highly expressed in both oocytes and pre-implantation embryos [21], [27], [54]. Moreover, a recent transgenic study using the oocyte-specific *Ctcf* RNAi approach [27] demonstrated its essential role in pre-implantation development suggesting that *Ctcf* may also be a maternal-effect gene. The authors reported that maternal depletion of CTCF resulted in both meiotic defects in oocyte development and mitotic defects in the embryo and that mitotic defects and apoptosis occurred at an earlier embryonic developmental stage (morula) than we observed (peri-implantation). This apparent discrepancy between the previous and our present work can be readily explained by the completely different technical approaches utilized in these studies. The previous RNAi-based approach would likely have efficiently depleted both maternal and zygotic *Ctcf* mRNA, likely resulting in earlier apoptosis. Thus these previous experiments did not clearly distinguish between the effects of maternal and zygotic expression of CTCF on pre-implantation development. Our present study, which utilizes a genetic knockout of the *Ctcf* locus, would not have affected the maternal *Ctcf* mRNA, which likely prolonged the viability of the embryo beyond the morula to the blastocyst stage. Thus our present observations clearly demonstrate that in the absence of the zygotic expression of CTCF, stable maternal *Ctcf* transcripts are sufficient to maintain pre-implantation development of the embryo, establishing *Ctcf* as a maternal-effect gene.

## Materials and Methods

### Ethics Statement

This study involved mice that were bred and housed in the centralized AAALAC accredited Fred Hutchinson Cancer Research Center Animal Facilities. All experimental procedures were performed in compliance with and approved by the Fred Hutchinson Cancer Research Center Institutional Animal Care and Use Committee (IACUC) protocol # 1271. Animals were euthanized humanely by carbon dioxide overdose as recommended by the Panel of Euthanasia of the American Veterinary Medical Association (AVMA).

### Chromosome mapping of the mouse *Ctcf* gene

The chromosomal location of *Ctcf* was determined by using the Jackson Laboratory interspecific backcross panels: (C57BL/6J×M. spretus) F1×C57BL/6J, called Jackson BSB, and (C57BL/6JEi×SPRET/Ei) F1×SPRET/Ei, called Jackson BSS [28]. A polymorphic region encompassing a simple sequence repeat located in the second *Ctcf* intron was used to distinguish between the C57BL/6J and the M. spretus *Ctcf* alleles. PCR primers used to amplify this region include: Forward: 5′ CCA GGA GAG CCA AGG ATA TAT AGT GAG ACC 3′ and Reverse: 5′ GGT TAG GAT TAC AGT GTA CAT CAC CAT ACC 3′ with a product size of 320 bp for the C57BL/6J *Ctcf* allele and 340 bp for the M. spretus allele.

### Targeted disruption of the *Ctcf* locus

The mouse *Ctcf* genomic locus was isolated from a 129sv mouse genomic library and overlapping lambda genomic clones encompassing all coding exons of *Ctcf* were mapped and partially sequenced. To make the targeting vector (Figure 2A) we employed a three-step ligation strategy. First the “left arm”, a 3.5 kb EcoR1 fragment from the lambda clone 4.12 was cloned into pZErO (Invitrogen), then the Not1-Apa1 fragment from the pPGKneobpaAlox2PGKDTA plasmid (from Phil Soriano) was cloned into pZero3.5EE, resulting in pleftArm plasmid. Finally the “right arm”, a 2.6 kb SpeI fragment from the lambda clone 13.2 was ligated into a compatible and unique Nhe1 site of the pLeftArm plasmid. This targeting construct harbors the PGK-neo cassette for positive selection and a PGK-DTA toxin expression cassette for negative selection. This linearized vector was electroporated into ES cells, and positive recombination was scored by Southern blots with the designated 5-prime and 3-prime probes located outside of the targeting cassette (Figure 2A, B). ES clones, positive for the wild type and mutated *Ctcf Eco*RV fragments, were selected to produce chimeric mice (Figure 2B). To excise the neo cassette, the *Ctcf* (+/−) heterozygous knockout mice were crossed to the MORE (Mox2Cre) mice, which express Cre recombinase from *Mox2* locus [30]. The resulting neo-excised *Ctcf* allele was documented by PCR using primers flanking the remaining LOX site as described under Genotyping. The heterozygous *Ctcf* (+/−) mice were backcrossed to 129sv genetic background. For embryonic lethality studies, the C57BL6/129sv F1 C*tcf* (+/−) mice were generated by crossing C57/BL6 *Ctcf* (+/+) mice to 129sv *Ctcf* (+/−) mice.

### FISH analysis

Metaphase chromosomes preparations were obtained from mice heterozygous for the *Ctcf* deletion and from control wild type mice. Spleens were dissected from the mice and short-term lymphocyte cultures were stimulated by lipopolysaccharide. Chromosome preparations were harvested using 0.75 M KCl and methanol: acetic acid (3∶1). Slides were denatured as described previously [55]. A 17 kb *Ctcf* genomic DNA fragment cloned in lambda phage (lambda 19.1, Figure 2A) was labeled with biotin by nick-translation using a Bionick kit from Invitrogen. A 170 kb BAC clone (MB11301, Research Genetics) used to mark the proximal region of mouse chromosome 8 was labeled with digoxygenin by nick-translation. Both probes were hybridized in the presence of mouse Cot1 DNA and of lambda phage DNA. Hybridization and washes were done as described previously [55]. The biotin-labeled *Ctcf* probe was revealed by an anti-biotin antibody made in goat followed by an anti-goat antibody labeled with fluorescein. A Cy3-labeled anti-digoxygenin antibody revealed the digoxygenin-labeled M11301 probe. Slides were stained with Hoechst and actinomycin D and signals examined by fluorescence microscopy.

### *Ex vivo* culture of pre-implantation stage embryos

All mouse studies were approved by the local Institutional Animal Care and Use Committee (IACUC) and all efforts were made to minimize suffering. *Ctcf* heterozygous intercrosses produced an average of 8–9 pre-implantation stage embryos per mating. All embryos collected were from natural matings without the use of hormone-stimulated super-ovulation. To isolate E0.5 one cell-stage embryos, mice on the day of cervical plug formation were sacrificed, and under an inverted microscope the egg sac was dissected from the oviduct and incubated briefly in M2 media (Specialty Media) plus hyaluronidase (100 ug/ml) to remove the cumulus layer as previously described [56]. The fertilized eggs were then isolated and cultured individually in 100 ul droplets of M16 media (Specialty Media) in 35 mm Petri dishes flooded with mineral oil. For the culture of E3.5 blastocyst stage embryos, mice were sacrificed three days after cervical plug formation and the uterus flushed with DMEM supplemented with 10% FBS. Under the inverted microscope, individual blastocysts were isolated and then cultured on pre-coated 0.1% gelatinized 6-well plates in DMEM, 10% FBS, and LIF (Leukaemia Inhibitory Factor, ESGRO, 1000 U/ml, Millipore) in a 37C incubator with 5% CO_2_. DNA and RNA was extracted from the freshly isolated or *ex vivo* cultured embryos.

### Genotyping and RT-PCR analysis of pre-implantation stage embryos

DNA and mRNA were simultaneously extracted from the freshly isolated or *ex vivo* cultured pre-implantation stage embryos using the modified DNA/RNA extraction protocol using the Dynal Dynabeads (Invitrogen). Briefly, pre-implantation stage embryos were collected in a drop of M2 media, rinsed in RNAse Away solution (Molecular Bioproducts) and then placed in 10 ul of the ‘cleared Dynabeads solution’ (DNA lysis buffer) for DNA processing according to the Dynabeads DNA Direct Universal kit protocol (Invitrogen). Following the lysis of the embryo and the DNA/Dynabeads complex formation using 170 ul of the resuspended Dynabeads per embryo, the supernatant was transferred to a clean, pre-chilled tube with 100 ul of the RNA lysis buffer from the Dynabeads mRNA Direct Kit (Invitrogen) and stored on ice for mRNA extraction until the DNA processing was complete. mRNA extraction was carried out using Oligo(dT)_25_ Dynabeads according to the mRNA Direct Kit protocol (Invitrogen).

For genotyping, the DNA was subjected to multiplex PCR utilizing primer sets recognizing the mutated and wild type *Ctcf* alleles. The primer pair for the wild type *Ctcf* allele is the following: Forward: 5′ GAG AAA GTA GTT GGT AAT ATG AAG CCT CC 3′ and Reverse: 5′ GGA CAT GTG TAA CTG CAA AGC TCA CAC TG 3′, with a product size of 420 bp. The primer pair identifying the knockout *Ctcf* allele includes: Forward: 5′ GGC ATG CTG GGG ATG CGG TGG GCT CTA TGG 3′ and Reverse: 5′ CCA GTG CCC TCT GAT ACA TGA TTG TGA TCC 3′, with a product size of 600 bp. Neo-excised allele genotyping primers are: Forward: 5′ TGA CCT AAC CCT AAC CCT AGC TGA 3′ and Reverse: 5′ TGA AAC TGA CTC TGA GCA AAG GGA 3′, with a product size of 516 bp.

For standard RT-PCR analysis the mRNA extracted from the embryos was reversed transcribed utilizing Superscript III (Invitrogen) under the following conditions. Random hexamers were added to 1–3 µg of RNA and incubated at 70°C for 5 minutes in a thermocycler. The temperature was cycled to 50°C and the RNA/primer mixture equilibrated for 5 minutes. Next, the reaction mixture (nuclease free H_2_O, 5× buffer, RNase inhibitor, dNTPs, DTT, and RT, except for no RT control), pre-equilibrated at 50°C, was added to the RNA/primer mixture and incubated for two hours at 50°C, followed by heat inactivation at 70°C for 5 minutes. *Ctcf* expression was analyzed using multiplex PCR conditions for both *Ctcf* and control *Gapdh* transcripts. Primers for the *Ctcf* transcript (GENBANK U51037.1) included: Sense: 5′ GAG CCT GCT GTA GAA ATT GAA CCT GAG CC 3′, (2188–2216) and Antisense: 5′ CCA ATA GTC CTG GTG CCG AGC AAG GCC CC 3′, (2551–2522) with a product size of 363 bp spanning *Ctcf* exons 11 and 12. Primers for the control *Gapdh* transcript included: Sense: 5′ CGT ATT GGG CGC CGT GTC ACC AGG GC 3′, and Antisense: 5′ GCC ATG AGG TCC ACC ACC CTG TTG CTG 3′, with a product size of 950 bp. RNA was tested for DNA contamination using a no-RT control and primers were verified by amplification of genomic DNA. The exact PCR conditions are available upon request.

For the real time RT-PCR analysis, cDNA was generated with SuperScript II reverse transcriptase (Invitrogen) under the following conditions. Random hexamers were added to 1.5 ug of RNA and incubated at 75°C for 5 min then transferred immediately to ice. Next, the reaction mix including 5× SuperScript II buffer, 0.1 M DTT, RNase inhibitor, dNTPs, and SuperScript II RT was added to the RNA/primer mixture and incubated at 45°C for 1 hour, then 50°C for 10 min, followed by heat inactivation at 75°C for 15 min. Real Time PCR analysis was performed on the automated ABI 7900 PCR machine (Applied Biosystems) using TaqMan Universal PCR Master Mix (Applied Biosystems) and a *Ctcf*-specific Taqman probe and primers spanning the junction between *Ctcf* exons 9 and 10 as follows: CTCF_mRNA_F1: 5′-TGC CTT TGT CTG TTC CAA GTG T-3′, probe: 5′-6FAM-ATT CAC CCG CCG GAA CAC AAT GGC A-NFQ-3′, mCTCF_mRNA_R1: 5′-CAG CAC AGT TAT CTG CAT GTC-3′. Amplification of *Gapdh* was performed in parallel reactions with primers and a Taqman probe as follows: forward primer: 5′-CCCGTAGACAAATGGTGAAGG-3′, probe: 5′- 6FAM-CGGTGTGAACGGATTTGGCCGTATT-3BHQ_1-3′, and reverse primer: 5′-AAATGGCAGCCCTGGTGA-3′. PCR was performed according to the manufacturer's instructions in 20 uL reactions, using 2 uL of 1∶1 dilution of cDNA from RT reaction described above. Standard ABI 7900 cycling conditions were followed. Sequence-specific amplification was detected by FAM (reporter dye) fluorescent signal during the amplification cycles. The standard curve assay (as described by Applied Biosystems) with serial dilutions of a standard was used for absolute quantification. Following quantification, *Ctcf*-mRNA levels were normalized to *Gapdh* expression. Each sample was assayed in triplicate; data represents mean +/− standard error.

### Immunofluorescence and TUNEL assay

Blastocysts were cultured on pre-coated 0.1% gelatinized glass slides or Lab-Tek permanox chamber slides (Nunc), fixed with 3.7% formaldehyde/PBS for 15 minutes and permeabilized with 0.5% Triton X-100/PBS at room temperature. The embryos were washed with PBS/PVP (3 mg/ml) and incubated with a blocking solution containing 10% NGS (ImmunoPure normal goat serum, Pierce Biotechnology)/PBS/0.1% Triton X-100 for 1 hour at room temperature followed by overnight incubation with a primary rabbit monoclonal anti-CTCF antibody (Cell Signaling) diluted 1∶300 in 2% NGS/PBS/0.1% Triton X-100 at 4°C. Following a PBS/PVP/0.1% Triton X-100 wash, the embryos were incubated with a secondary goat Texas red-conjugated anti-rabbit antibody (1∶200) (Jackson Laboratory) for 1 hour at room temperature. After washing, slides were counterstained with DAPI. Apoptosis was assessed using the DNA fragment end labeling kit (FragEL, Calbiochem) following the manufacturer's instructions.
